# Diselenides and Benzisoselenazolones as Antiproliferative Agents and Glutathione-S-Transferase Inhibitors

**DOI:** 10.3390/molecules24162914

**Published:** 2019-08-11

**Authors:** Dorota Krasowska, Nunzio Iraci, Claudio Santi, Józef Drabowicz, Marcin Cieslak, Julia Kaźmierczak-Barańska, Martina Palomba, Karolina Królewska-Golińska, Jakub Magiera, Luca Sancineto

**Affiliations:** 1 Division of Organic Chemistry, Centre of Molecular and Macromolecular Studies, Polish Academy of Science, Sienkiewicza, 112, 90-363 Lodz, Poland; 2 Department of Pharmacy, University of Salerno, Via G. Paolo II 132, 84084 Fisciano, Salerno, Italy; 3 Department of Pharmaceutical Sciences, University of Perugia, Via del Liceo 1, 06123 Perugia, Italy; 4 Institute of Chemistry Jan Długosz University in Częstochowa Częstochowa, 42-200 Armii Krajowej 13/15, Poland; 5 Division of Bioorganic Chemistry, Centre of Molecular and Macromolecular Studies, Polish Academy of Science, Sienkiewicza, 112, 90-363 Lodz, Poland

**Keywords:** diselenide, benzisoselenazolone, breast cancer, glutathione S-transferase

## Abstract

A series of variously functionalized selenium-containing compounds were purposely synthesized and evaluated against a panel of cancer cell lines. Most of the compounds showed an interesting cytotoxicity profile with compound **5** showing a potent activity on MCF7 cells. The ethyl amino derivative **5** acts synergistically with *cis*-platin and inhibits the GST enzyme with a potency that well correlates with the cytotoxicity observed in MCF7 cells. A computational analysis suggests a possible binding mode on the GST enzyme. As the main outcome of the present study, the ethyl amino derivative **5** emerged as a valid lead compound for further, future developments.

## 1. Introduction

Cancers and neoplastic disorders are global health threats due to a year-on-year increase in the number of new diagnoses and deaths. The International Agency for Research on Cancer (IARC) estimates that one-in-five men and one-in-six women worldwide will develop cancer, and that one-in-eight men and one-in-eleven women with a history of cancer will die from their disease [1]. Just to give a brief overview, according to the World Health Organization (WHO) cancer is the second leading cause of death globally, with an estimated 9.6 million deaths and more that 18 million of new cases in 2018. Among women, the most frequent cancer, impacting 2.1 million individuals yearly, is breast cancer. It also causes the greatest number of cancer-related deaths. Besides, with an estimated 570,000 new cases in 2018, cervical cancer is the fourth most frequent cancer in women, representing 6.6% of all female cancers [2,3]. Similarly to solid tumors, blood cancers are pandemic; indeed, approximately every three minutes, one person in the United States (US) is diagnosed with leukemia or lymphoma and one death every nine minutes is because of these neoplasms [4].

Globally, cancer treatments have an estimated total economic cost of 1 trillion US dollars [5]. The use of cytotoxic agents, together with radiation therapy, is still the first line of treatment for most of solid and blood cancers. The so-called cancer chemotherapy consists of the simultaneous administration of drugs, endowed with heterogeneous mechanisms of actions, that synergistically interact in a clear attempt to cope with the clinical problem of cancer multi drug resistance (MDR) and tumor cell heterogeneity [6]. Unfortunately chemotherapy is linked with serious side effects that affect the quality of life of patients in earnest. Most of the chemotherapeutic drugs, with very few exceptions, are highly reactive and unselective compounds that target both normal and cancer cells. While normal cells are capable of fixing the damage eventually created by chemotherapeutics, cancer cells are not, thus making them more susceptible to the cytotoxicity [7,8].

**Figure 1:**
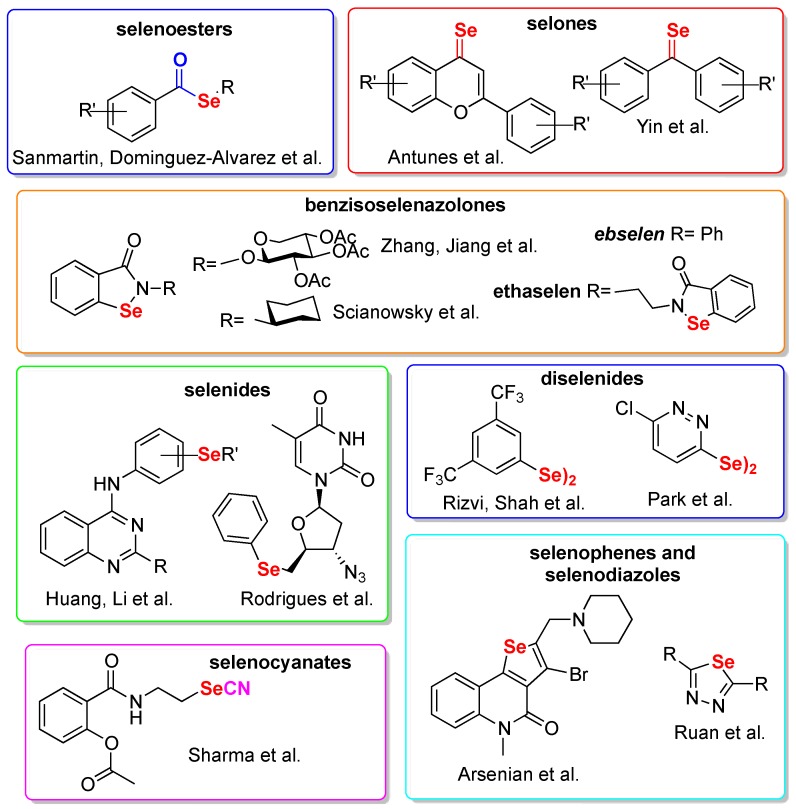
Selection of Se-containing compounds preclinically profiled as anti-neoplastic agents.

Selenium (Se) is an essential nutritional trace element and its deficiency correlates with various human diseases [9]. Its incorporation into organic molecules to develop bioactive compounds has only recently been adopted [10,11] since, for more than half of its history, selenium has been considered as a poison [12,13]. Starting from the identification of ebselen [14] (Figure 1) as a mimic of the key antioxidant enzyme glutathione peroxidase [15], medicinal chemistry efforts have led to the discovery of several selenium-containing compounds worthy of mention, such as ebselen, currently under clinical investigations for a number of disorders [11,16], or ethaselen, evaluated for the treatment of thioredoxin reductase overexpressing non-small cell lung cancers [17]. The lesser known selenazine derivative ALT2017 (not shown), was recently investigated for the treatment of diabetes and psoriasis [18]. Besides, several interesting examples of preclinical compounds have been reported, such as the anti-HIV DiSeBAs [19], the sole selenium-containing NCp7 inhibitor reported to date [20,21], the selenides carbonic anhydrase inhibitors reported by Capperucci and Supuran [22], and the ebselen-like compounds able to inhibit bacterial urease [23], just to quote some among the most recent examples.

Being selenorganic compounds redox active agents, they are particularly fit for the development of anticancer chemotherapeutics. Cancer cells are indeed characterized by the so-called Warburg effect, i.e., an increased aerobic glycolysis that leads to high basal levels of reactive oxygen species [24]. In addition, Se and selenium-containing compounds have proved to be selectively absorbed by cancer cells [25] and have been shown to alter cancer angiogenesis [26,27]. This body of literature represents a strong rationale and gives reason to the number of cancer-targeting selenium-containing compounds reported in literature. Most of them were recently reviewed by some of us [28], by Fernandez [29], and by Domínguez-Álvarez [30], and a selection of these compounds is presented in Figure 1.

Selenoesters were reported by Sanmartin et al. in a series of papers where their synergistic activity with marketed drugs was also tested [31,32]; Antunes and Yin converted the keto functionality of flavonoids and isocombretastatins into selone getting compounds endowed with in vivo antineoplastic activities [33,34]. In vivo data are also available for the selenocyanate-containing aspirin analogue developed by Sharma and coworkers [35]. The selenide functional group was exploited by Rodriguez and Huang for the development of AZT analogues and quinazoline-based compounds, respectively [36,37]. Zhang et al. [38] and Scianowski et al. [39] took inspiration from ebselen, while the diselenide functional group was fairly explored [40,41]. Arsenian et al. developed some selenopheno quinolinones [42] and, finally, the most recent paper in chronological order reports selenodiazole analogues bioavailable enough to be tested in vivo in a model of liver tumor [43].

Glutathione *S*-transferases (GSTs) are a widely distributed family of enzymes with diverse biological roles, including cell defense against oxidative stress and toxic molecules. These enzymes exert protection by conjugating glutathione (GSH) to a wide variety of substrates the cells judge toxic, making them suitable for further modification, and finally excreting them from the cellular environment [44]. In cancer cells, GSTs are often overexpressed, leading to an increased detoxification from anticancer drugs [45]. GSTs overexpression, together with efflux pumps, is the main contributor in cancer MDR [45]. GST overproduction in cancer cells offers an opportunity for the selective activation of prodrugs leading to high concentrations of the active principle in neoplastic districts [46]. Taken together, these results make GST a suitable, yet poorly exploited, target for the development of anticancer compounds or agents that act synergistically with known, marketed drugs.

In this paper we report the design and synthesis of selenium containing compounds, and their detailed biological evaluation on cancerous and non-cancerous cellular lines. The cytotoxicity of the best-in-class compounds was evaluated in combination with two marketed drugs and the anti-GST activity was tested. In conclusion, a computational analysis was carried out to shed light on the possible compound-GST interaction.

## 2. Results

**Figure 2:**
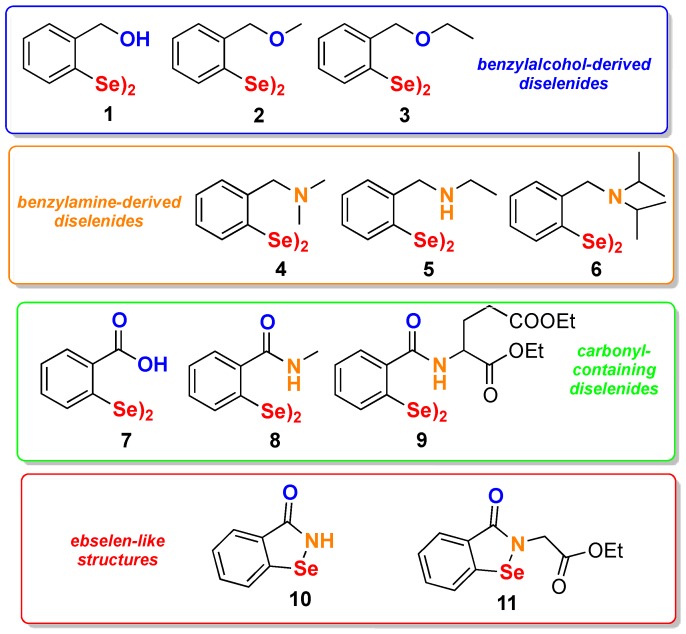
Collection of compounds selected for this study.

A series of selenium-containing compounds was designed, synthesized, and then tested for their influence on the proliferation of different cancer cell lines. As reported in Figure 2, four groups of derivatives can be identified. In the benzyl alcohol diselenide (**1**), an intramolecular Se--O weak interaction (chalcogen bond), which proved to deeply influence the biological properties of Se-containing compounds [47,48], was present; in addition, an increased steric hindrance was introduced in compounds **2** and **3**. A different nonbonded interaction was introduced in compounds **4**–**6** where a disubstituted or a monosubstituted amine was placed in *ortho* position to the selenium. Carbonyl-containing diselenides **7**–**9** were included in this study. Beside compound **7**, which had already been reported by us with its hormetic activity [49], amides **8** and **9** were purposely synthesized in order to check the effect of this decoration on the anticancer activity. Finally, compounds containing the benzisoselenazolone scaffold were prepared by taking inspiration from *ebselen* and *ethaselen*, the latter being the most studied selenium-containing compound in anticancer research [17,50].

### 2.1. Synthesis

**Scheme 1:**
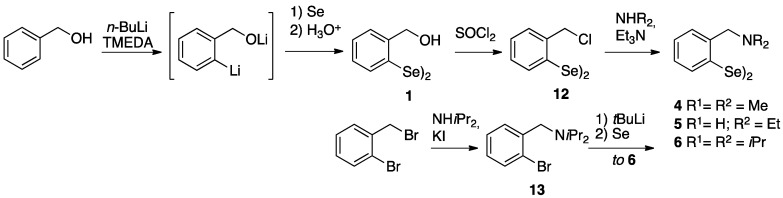
Synthesis of compound **1** and its further transformations.

As reported in Scheme 1, compound **1** was synthesized through a two-step process starting from benzyl alcohol initially *ortho* lithiated with *n*-butyllithium in dry pentane. The lithium salt was then treated with elemental Se to give the corresponding selenol, that spontaneously converted into the target diselenide after oxidative work-up [51]. Compound **1** was the starting compound for the preparation of two amino derivatives, i.e., compound **4** and **5**, through its conversion into the chloride **12,** then submitted to nucleophilic displacement in the presence of triethylamine as HCl scavenger. The use of sterically demanding diisopropylamine as nucleophile under the same reaction condition was found unsuccessful, thus compound **6** was obtained in low yield. As a result, a different approach was developed in order to improve the yield. It consisted in the preparation of *ortho*-bromo *N,N*-diisopropylbenzylamine followed by the metal-halogen exchange and subsequent addition of selenium. In this way compound **6** was obtained in 64% yield.

**Scheme 2:**
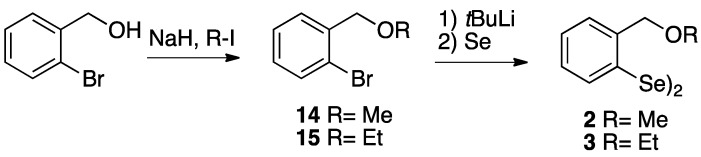
Synthesis of the ethers **2** and **3**.

Methyl and ethyl ether derivatives, **2** and **3** respectively, were achieved following the reaction sequence illustrated in Scheme 2. Starting from the commercially available *ortho*-bromobenzyl alcohol, an initial alkylation followed by the metal-halogen exchange and subsequent addition of elemental selenium afforded the ether derivatives in good yields.

**Scheme 3:**
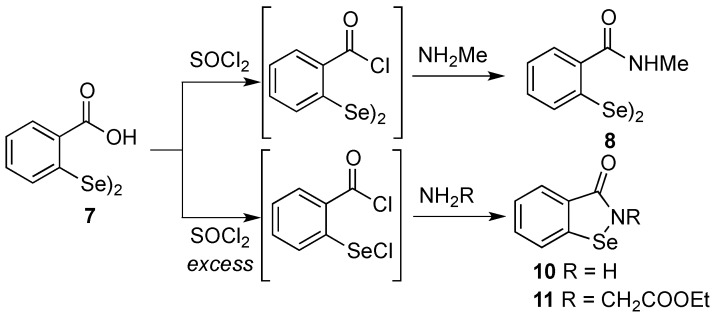
Synthesis of the ethers **8**, **10,** and **11**.

While compound **7 [52,53]** and **9 [19]** were synthesized according to previously reported procedures, compounds **8**, **10** and **11** were prepared following the synthetic path illustrated in Scheme 3. In particular, by treating the benzoic acid diselenide **7** with stoichiometric amounts of thionyl chloride the diselenide bridge was preserved and the acidic chloride was selectively obtained. It was not isolated but directly reacted with pure methylamine, giving compound **8** in good yield and purity. Conversely, the ebselen-like compounds **10** and **11** were obtained by using SOCl_2_ in ~3 molar excess in the respective reactions in Scheme 3, after the reaction with ammonia or ethyl glycinate.

### 2.2. Pharmacological Evaluation

#### 2.2.1. Cytotoxicity Screening

**Figure 3:**
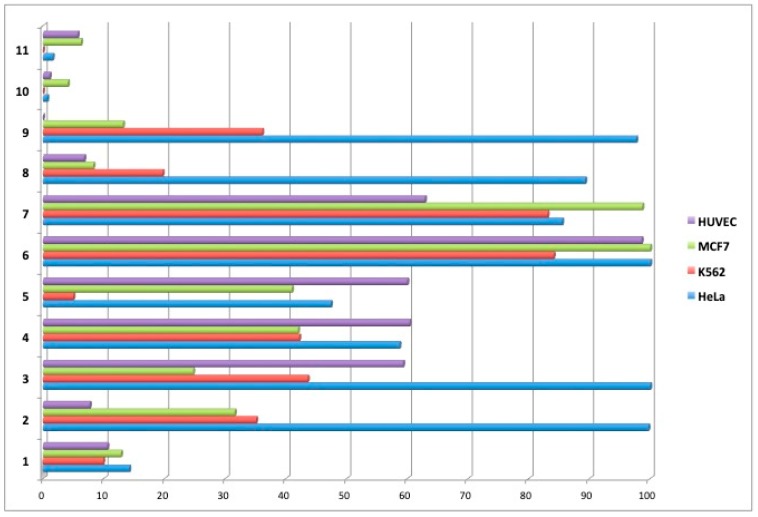
Cell survival [%] after 48 h incubation with test compounds at 100 μM.

Selenium-containing compounds were screened for their cytotoxic properties using MTT assays. The following cell lines were used: K562 (leukemia), HeLa (cervix carcinoma), MCF7 (breast cancer), and non-cancerous HUVEC (human umbilical vein endothelial). Cells treated with 1% DMSO served as a control (100% viability). Initially, the viability of cells was determined after 48 h incubation with compounds at the concentration of 100 µM, the results are summarized in Figure 3. Unexpectedly, we noticed that compounds **4** and **5** were not compatible with the MTT assay because after addition of tetrazolium salt, they caused a concentration-dependent color development. Therefore, the MTT assay was modified for these two compounds, i.e., after 48 h incubation with adherent cells (HeLa, MCF7) and just before the addition of tetrazolium salt, the cell medium was replaced with a fresh one that did not contain **4** or **5**. In the case of suspension K562 cells, the viability and IC_50_ measurements for **4** and **5** were done using ATP-based luminescence assay (see materials and methods for details).

Compounds that displayed significant cytotoxicity at 100 μM (i.e., reduced cancer cells survival by at least 50%) were further tested at different concentrations. Based on the obtained data, IC_50_ values were calculated and are reported in Table 1.

#### 2.2.2. Inhibition of Glutathione *S*-Transferase (GST) Activity

**Figure 4:**
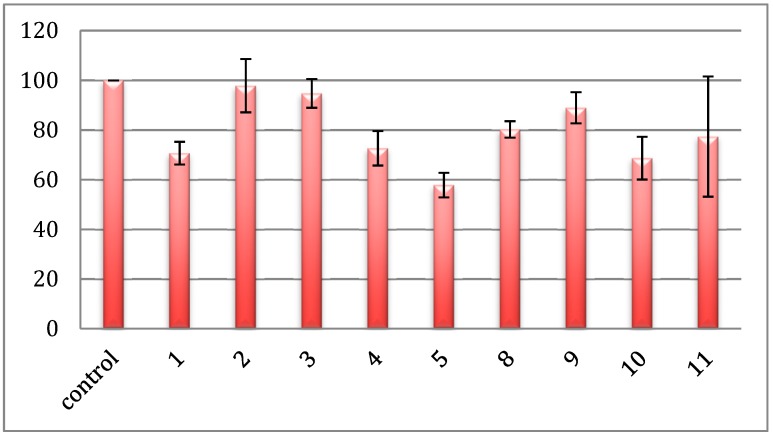
Anti-GST activity of test compounds at the concentration of 10 µM.

Glutathione S-transferases (GSTs) comprise a large group of enzymes involved in the removal of xenobiotic from cells [44,46]. GSTs catalyze conjugation of glutathione with electrophilic substrates (for example carcinogens, environmental pollutants, or anticancer drugs), which are subsequently pumped out of the cell. Based on reports [28,49], we hypothesized that the selenorganic compounds herein reported might inhibit the activity of GST. Therefore, we measured the activity of purified equine liver GST in the presence of test compounds at the concentration of 10 µM. Figure 4 summarizes the inhibitory effect of compounds on the activity of GST. Interestingly, some of them exerted a moderate inhibition. This is particularly evident for compounds **1**, **10,** and **5**, which reduced the enzymatic activity by 30–40% (Figure 4).

### 2.3. Molecular Docking Simulations

**Figure 5:**
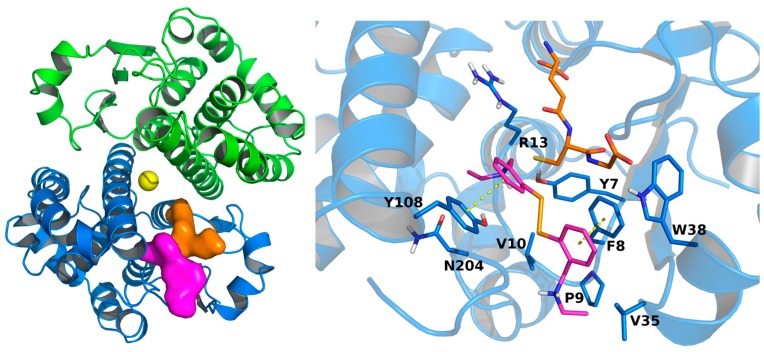
**Left**, docking-predicted binding site for compound **5** (Magenta surface). GST crystal structure is depicted in blue (chain A) and green (chain B) cartoons. Glutathione (PDB ID: 5DJM) and cis-platin (PDB ID: 1AQW) experimental coordinates are represented in orange and yellow, respectively. **Right**, docking-predicted bound conformation of compound **5**, in magenta sticks. GST crystal structure is depicted in blue sticks and cartoons. Glutathione experimental bound conformation (PDB ID: 5DJM) is depicted in orange sticks. Potential π–π interactions are represented by yellow dashed lines.

Given its anti-GST activity, compound **5** was selected for molecular docking simulations meant to investigate its interaction with the enzyme. The compound was flexibly docked onto the whole apo GST structure (PDB ID: 5DCG) using Autodock Vina [54]. Compound **5** docked into the H-site, embraced by residues Y7, F8, P9, V10, G12, R13, V35, W38, I104, Y108, N204, and G205 (Figure 5). Both amine moieties of compound **5** may engage in H-bond interaction with the protein. One is indeed in the proximity of the hydroxyl groups of Y7 and Y108 (N-O distances 3.09Å and 4.19Å, respectively), while the other one lies at 3.17Å from the backbone oxygen of G205. The docked conformation shows that the geometry of the diselenide bridge permits the two aromatic rings to engage in π–π stacking interactions with Y108 and F8. π–π stacking interactions at the H-site are pretty common among H-site binders, as they are found in several co-crystal structures [55,56,57].

### 2.4. Synergistic Studies with Doxorubicin and Cisplatin.

**Figure 6:**
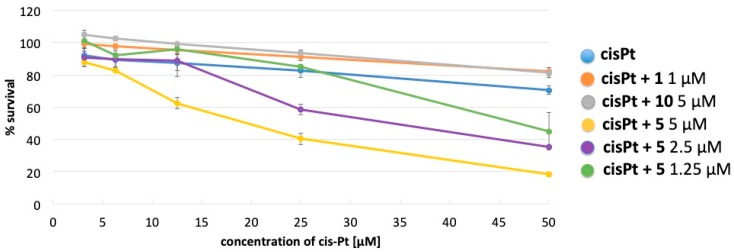
Toxicity of cis-Pt in presence of compounds **1**, **5,** and **10**.

Cisplatin (cis-Pt) and doxorubicin are widely used in the treatment of many cancers. These drugs are metabolized with the aid of GSTs, resulting in their efficient removal from cells. Indeed, cancer cells very often overexpress GSTs and the therapeutic effect of cis-Pt or doxorubicin may be significantly limited. This limitation could be overcome by the use of GST inhibitors. We examined whether inhibition of GST by compound **1**, **10,** or **5** would increase the toxicity of cis-Pt or doxorubicin. K562 cells were treated with increasing concentrations of cis-Pt or doxorubicin in the presence of compound **1** (1 µM), **10** (5 µM), or **5** (5 µM) for 48 h. At these concentrations, the selenorganic compounds had no effect on the survival of K562, only compound **5** at 5 µM slightly reduced K562 survival to 85–90% (data not shown). As shown in Figure 6, the cytotoxicity of cis-Pt against K562 cells was significantly enhanced in the presence of **5**, while **1** and **10** did not show any effect. Moreover, as shown in Figure 5, the effect of **5** seems to be concentration dependent. On the other hand, none of the tested compounds enhanced the cytotoxicity of doxorubicin in K562 cells (data not shown).

## 3. Discussion

A series of a selenium-containing compounds was prepared through different methodologies allowing the achievement of the target compounds in moderate to good yields. Looking at the synthetic strategies, it is possible to highlight that the use of selenium as electrophile is the method of choice for the preparation of aromatic diselenide bearing electron donating groups in *ortho* position to the selenium (see Scheme 1 and Scheme 2). On the contrary, when an electron-withdrawing group is present in the aromatic ring, the selenium containing functional group is better introduced as nucleophile. Once obtained, some of the diselenides were functionalized through late stage reactions (see compound **4**, **5,** and **8**) while for compounds **2**, **3,** and **6** selenium installation was the last reaction step, highlighting that the synthetic tractability of some diselenides is still to be improved through the development of novel synthetic methodologies.

All the selenium-containing compounds were assayed for their cytotoxic activity towards three different cancer cell lines and non-cancerous HUVEC cells in a two-step approach. Initially, all of the compounds were screened at the fixed concentration of 100 µM; then, for those displaying a significant antiproliferative activity, the IC_50_ was determined. From a structure activity relationship (SAR) standpoint, the benzisoselenazolone scaffold confirms its cytotoxic properties, here displayed by compounds **10** and **11**, endowed with a good and wide spectrum activity, which is however not selective, being the compounds able to inhibit the proliferation of the normal HUVEC cells. The cytotoxicity is not influenced by the substituent on the amidic nitrogen because both compounds are equivalently potent in terms of IC_50_, while, when looking at the GST inhibitory activity the amidic substitution seems to play a role, as **10** is slightly more potent than **11**. Among diselenides, compound **7** confirmed, in this experimental setting, its lack of antiproliferative activity as we previously reported [49]. Benzyl alcohol-derived diselenides (compounds **1**–**3**), gave intriguing SAR information; indeed all of them displayed an unselective activity but their potency decreases as the steric hindrance on the benzylic oxygen increases (**1** OH > **2** OMe > **3** OEt). Compound **1** confirmed its therapeutic potential as it was previously tested by Ali Shah, although on different cancer cell lines [40]. Worthy to be mentioned, compound **3** showed a moderate activity on MCF7 cells coupled with the lack of toxicity on normal HUVEC cells (Table 1, entry 3). The steric hindrance is also important for the anti-GST activity where compound **1** is yet the most potent. In this regard the presence of a H-bond donor is plausibly important for the activity as demonstrated further in the amine series (compounds **4**–**6**). In this series, while the isopropyl amino derivative **6** was inactive, compounds **4** and **5** displayed low micromolar potency against the three cancer cell lines tested. As mentioned above, we found some incompatibility between the MTT assay and compounds **4** and **5,** that initially led to an underestimation of their activity and that required a slight modification of the assay protocol. This finding should be taken into account in future investigation employing the MTT method on amino group-containing diselenides. Among this series, the sole compound **5** inhibited GST activity to a relevant extent at 10 µM.

*Ortho*-amidic diselenides **8** and **9** were inactive in HeLa cells while showing an interesting activity on leukemia and breast cancer cellular models but none of them were able to block GST activity.

When tested in combination with cis-Pt and doxorubicin on K562 cells, the GST inhibitors **1**, **5**, and **10** gave contrasting results. None of them were found to exert any synergistic effect with doxorubicin, which is however potent enough to reasonably conceal the synergistic effect of the compounds. On the opposite, compound **5** exerted a dose dependent synergism with cis-Pt. Indeed, when tested at 5 µM it significantly increased the drug toxicity. At this concentration the cytotoxicity of **5** was minimal, and the observed effect cannot be seen as additive. A plausible explanation entails the inhibition of GST by compound **5,** which in turn hampers the removal of cis-Pt from cancer cells. Worthy to be mentioned, the anti GST activity of the amino derivative **5** correlates well with the anti-cancer activity observed in MCF7 cells. Synergistic studies between selenium-containing compounds and known antineoplastic drugs are published in literature [31], but just a few examples report an improvement of cis-Pt potency [58,59], and compound **5** represents the first diselenide which carries out this sort of talent. The combination of selenorganic compounds with cis-Pt is surely fruitful; cis-Pt indeed exerts, beside the therapeutic response, severe side effects such as the ototoxicity, which seems to be attenuated by selenium sources, as demonstrated for ebselen [60].

The computational analysis, carried out using the crystal structure of GST as a target, clearly indicates that the compound might be hosted in the H-site of the enzyme active site stabilized by multiple interactions. Noteworthy, the diselenide bridge docks close to the Y7 phenol group, which has been proven to be critical for GST catalytic activity [61,62,63]. In addition, **5** engages in π–π stacking interaction with Y108, which has been proven to be critical for the enzymatic activity [61] as well as a target of covalent inhibition [64,65].

## 4. Materials and Methods

### 4.1. Cells and Cytotoxicity Assay

The HeLa (human cervix carcinoma) and K562 (leukemia) cells were cultured in RPMI 1640, while MCF7 (breast carcinoma) was cultured in MEM medium. The media were supplemented with antibiotics and 10% fetal calf serum. Cells were grown in a 5% CO_2_–95% air atmosphere. For the cytotoxicity assay, 7 × 10^3^ cells were seeded on each well on 96-well plate (Nunc, Roskilde, Denmark). Human umbilical vein endothelial cells (HUVEC) were from Life Technologies and cultured in Medium 200 with low serum growth supplement (Life Technologies, Waltham, MA, USA) according to manufacturer’s instructions. 10 × 10^3^ cells were seeded on each well on a 96-well plate (Nunc). 24 h later cells were exposed to the test compounds for additional 48 h. Stock solutions of test compounds were freshly prepared in DMSO. The final concentrations of the compounds tested in the cell cultures were: 2 × 10^−1^ mM, 1 × 10^−1^ mM, 5 × 10^−2^ mM, 1 × 10^−2^ mM, 1 × 10^−3^ mM, 1 × 10^−4^ mM. The concentration of DMSO in the cell culture medium was 1%.

The cytotoxicity of compounds was determined by the MTT [3-(4,5-dimethylthiazol-2-yl)-2,5-diphenyltetrazolium bromide; Sigma, St. Louis, MO, USA] assay as described [66]. Briefly, after 48 h of incubation with compounds, the cells were treated with the MTT reagent for 2 h. MTT-formazan crystals were dissolved in 20% SDS and 50% DMF at pH 4.7 and absorbance was read at 570 and 650 nm on the FLUOstar Omega plate reader. Cells grown in the presence of 1% DMSO were used as a control (100% viability). Since **4** and **5** interfered with MTT assay, their toxicity in HeLa and MCF7 cells was measured as above except that after 48 h incubation and just before the addition of MTT, the cell medium was replaced with a fresh one without compounds. In the case of K562 cells, the cytotoxicity of **4** and **5** was measured with CellTiter-Glo luminescent cell viability assay (Promega) according to manufacturer’s instructions. The luminescence was measured using FLUOstar Omega plate reader (BMG Labtech, Offenburg, Germany).

The values of IC_50_ (the concentration of test compounds required to reduce the cell survival to 50% of the control) were calculated from dose-response curves and used as a measure of cellular sensitivity to a given treatment.

The effect of **1** (at 1 µM), **10** (5 µM), and **5** (at 1.25, 2.5 or 5 µM) on the toxicity of cis-Pt or doxorubicin was measured in K562 cells. The concentrations of cis-Pt were 3.12, 6.25, 12.5, 25, 50 µM and those of doxorubicin were 0.15, 0.312, 0.625, 1.25, 2.5, 5 µM. Test compounds (or DMSO as vehicle control) and cis-Pt or doxorubicin were added to K562 cells simultaneously. Cells viability was measured 48 h later by CellTiter-Glo luminescent cell viability assay (Promega).

### 4.2. GST Activity Assay

The inhibitory activity of organoselenium compounds against equine liver GST was tested using Glutathione S-Transferase Assay Kit from Cayman Chemical (item 703302, Ann Arbore, MI, USA). All reagents were provided in the assay kit: GST enzyme (lyophilized), assay buffer, glutathione (GSH) and CNDB (1-chloro-2,4-dinitrobenzene). Lyophilized GST enzyme was reconstituted in water immediately prior use according to manufacturer’s instructions. The final reaction volume was 100 µL. The samples contained 74 µL assay buffer, 10 µL GSH, 10 µL GST enzyme, 1 µL of test compound (final concentration 10 µM) or 1 µL DMSO (control) and 5 µL CNDB. Blank sample contained 85 µL assay buffer, 10 µL GSH and 5 µL CNDB. Immediately after addition of CNDB the absorbance at 340 nm was read (time zero) and subsequently every 2 min for total time of 20 min. The readings were taken by FluoStar Omega plate reader in a kinetic mode. The activity of GST enzyme in a control sample was calculated and expressed as 100% and used for calculation of relative inhibition by the test compounds.

### 4.3. Chemistry

Reactions were conducted in round bottom flask and were stirred with Teflon-coated magnetic stirring bars. Commercially available organic and inorganic reagents were used without further purification unless otherwise stated. Tetrahydrofuran, diethyl ether, dioxane were dried over sodium and distilled under argon. Dichloromethane was dried over calcium hydride and distilled under argon prior to use. Analytical thin-layer chromatography (TLC) was performed on silica gel 60 F_254_ precoated aluminum foil sheets and visualized by UV irradiation or by KMnO_4_ staining followed by gentle heating. Silica gel Kieselgel 60 (70–230 mesh) was used for column chromatography. NMR experiments were conducted at 25 °C with a Bruker Avance 200 spectrometer (Billerica, MA, USA) operating at 200.16 MHz for ^1^H, or with a Bruker Avance III 500 spectrometer operating at 500.13 MHz for ^1^H, 125.76 MHz for ^13^C and 95.43 MHz ^77^Se experiments. ^1^H, ^13^C and ^77^Se chemical shifts (δ) are reported in parts per million (ppm), and are cited with respect to TMS (δ = 0.0 ppm), as internal standard (^1^H) and the residual solvent peak of CDCl_3_ (δ = 7.26 and 77.00 ppm in ^1^H and ^13^C-NMR, respectively) or Ph_2_Se_2_ (^77^Se δ = 463 ppm) as the external standard. Data are reported as: Chemical shift (multiplicity, coupling constants where applicable, number of hydrogen atoms, and assignment where possible). Abbreviations are: s (singlet), d (doublet), t (triplet), q (quartet), dd (doublet of doublet), dt (doublet of triplet), tt (triplet of triplet), m (multiplet), br. s (broad signal). Coupling constant (*J*) quoted in Hertz (Hz) to the nearest 0.1 Hz. High-resolution mass spectrometry (HRMS) measurements were performed using Synapt G2-Si mass spectrometer (Waters, Milford, MA, USA) equipped with an APCI source and quadrupole-Time-of-Flight mass analyzer. The mass spectrometer was operated in the positive and negative ion detection modes with discharge current set at 4.0 μA. The heated capillary temperature was 350 °C. The results of the measurements were processed using the MassLynx 4.1 software (Waters) incorporated with the instrument. Melting points (m.p.) were determined on Mel-Temp^®^ apparatus (Electrothermal, Rochford, Essex, UK) and are uncorrected.

#### 4.3.1. Bis[2-(hydroxymethyl)phenyl] Diselenide (**1**)

The synthesis of di(2-hydroxybenzyl) diselenide was prepared according to the procedure reported in [51]. Yield (64%). ^1^H-NMR (CDCl_3_) δ: 1.80 (br. s, 1H, O*H*), 4.72 (s, 2H, C*H_2_*OH), 7.20 (dt, *J =* 1.4 and 7.6 Hz, 1H, Ar*H*), 7.30 (dt, *J =* 1.4 and 7.3 Hz, 1H, Ar*H*), 7.39 (dd, *J =* 1.3 and 7.5 Hz, 1H, Ar*H*), 7.68 ppm (dd, *J =* 1.2 and 7.7 Hz, 1H, Ar*H*); ^13^C-NMR (CDCl_3_) δ: 65.40, 128.43, 128.84, 128.95, 130.66, 135.03, 142.10 ppm; ^77^Se NMR (CDCl_3_) δ: 431.74 ppm. HRMS calculated for C_14_H_14_O_2_Se_2_Na^+^ = 396.9222, found =396.9224.

#### 4.3.2. Bis[2-(chloromethyl)phenyl] Diselenide (**12**)

Compound **12** was prepared in 83% yield from compound **1** by using of thionyl chloride as described in the literature [67]. ^1^H-NMR (CDCl_3_) δ: 4,67 (s, 2H, C*H_2_*Cl), 7.23, (dt, *J =* 1.5 and 7.6 Hz, 1H, Ar*H*), 7.29 (dt, *J =* 1.2 and 7.4 Hz, 1H, Ar*H*), 7.38 (dd, *J =* 1.2 and 7.6 Hz, 1H, Ar*H*), 7.72 ppm (dd, *J =* 1.0 and 7.6 Hz, 1H, Ar*H*); ^77^Se NMR (CDCl_3_) δ: 437.97 ppm.

#### 4.3.3. Bis[2-(*N*,*N*-dimethylaminomethyl)phenyl] Diselenide (**4**)

To a solution of bis[2-(chloromethyl)phenyl] diselenide **12** (0.293 g, 0.73 mmol) in THF (4 mL) were added triethylamine (0.2 mL, 1.45 mmol) and 2M solution of dimethylamine in THF (1.5 mL, 2.92 mmol) under argon atmosphere. The reaction mixture was stirred at 36 °C for 24 h and then concentrated under reduced pressure. The mixture was diluted with Et_2_O and the ethereal solution was extracted with a 1% aqueous solution of hydrochloric acid (2 × 20 mL). The combined acidic aqueous layers were alkalized with solid K_2_CO_3_, and extracted with dichloromethane (3 × 20 mL). The organic layers were combined, washed with brine, dried over anhydrous magnesium sulfate and evaporated under reduced pressure. The obtained crude product was purified by silica gel column chromatography (hexane-ethyl acetate 10:0.5) to afford product **4** (0.209 g, 67%) as a yellow oil. ^1^H-NMR (CDCl_3_) δ = 2.27 (s, 6H, C*H_3_*), 3.56 (s, 3H, C*H_2_*), 7.09–7,14 (m, 3H, Ar*H*), 7.77–7.82 ppm (m, 1H, Ar*H*); ^13^C-NMR (CDCl_3_) δ = 44.39, 64.64, 125.83, 128.16, 128.47, 131.42, 134.30, 139.15; ^77^Se NMR (CDCl_3_) 428.24 ppm; HRMS calculated for [C_18_H_25_N_2_Se_2_^+^] = 429.0348, found = 429.0348.

#### 4.3.4. *N*,*N*′-((Diselanediylbis(2,1-phenylene))bis(methylene))diethanamine (**5**)

To a solution of bis[2-(chloromethyl)phenyl] diselenide **12** (0.558 g, 1.38 mmol) in THF (15 mL) trietylamine (0.38 mL, 2.76 mmol) and ethylamine (2 M in THF, 2.8 mL, 5.55 mmol) were added dropwise under argon atmosphere. The reaction mixture was stirred at 50 °C for 2 days. After the reaction was complete, the mixture was concentrated under reduced pressure. The resulting oil was diluted with Et_2_O and the ethereal solution was extracted with a 1 M aqueous solution of hydrochloric acid (3 × 20 mL). The combined acidic aqueous layers were alkalized with solid K_2_CO_3_, and extracted with dichloromethane (2 × 20 mL). The organic layers were combined, washed with brine, dried over anhydrous magnesium sulfate and the solvent was evaporated in a vacuum. The product was purified by silica gel column chromatography (DCM-methanol 25:1 then DCM-methanol 9:1) and isolated as yellow oil (0.371 g, 63% yield). ^1^H-NMR (CDCl_3_) δ = 1.12 (t, *J =* 7.1 Hz, 3H, C*H_3_*), 2.68 (q, *J =* 7.1 Hz, 2H, C*H_2_*CH_3_), 3.30 (s, 1H, N*H*), 3.92 (s, 2H, C*H_2_*N), 7.10–7.23 (m, 3H, Ar*H*), 7.74–7.79 ppm (m, 1H, Ar*H*). ^13^C-NMR (CDCl_3_) δ = 13.96, 42.82, 52.80, 127.50, 128.77, 129.06, 133.18, 133.50, 138.12; ^77^Se NMR (CDCl_3_) δ = 422.54 ppm; HRMS calculated for [C_18_H_25_N_2_Se_2_^+^] 429.0348, found = 429.0358.

#### 4.3.5. 2-Bromo-*N*,*N*-diisopropylbenzylamine (**13**)

A slight modification of the previously reported procedure [68] was applied. A round bottom flask equipped with stirring bar was charged with potassium iodide (31.3 mg, 0.19 mmol) and the solution of 2-bromobenzyl bromide (0.471 g, 1.88 mmol) and diisopropylamine (0.65 mL, 7.54 mmol) in 2 mL of dry dioxane was added. After being stirred at room temperature for 3 days under argon atmosphere, the flask was fitted with the condenser and the reaction mixture was heated to 50 °C with continuous stirring for 2 days. An aqueous solution of sodium bicarbonate (10 mL) and diethyl ether (10 mL) were added to the cooled reaction mixture and the solution was extracted. The combined ethereal phases were washed with brine, dried over anhydrous magnesium sulfate, filtered and concentrated in vacuo to give product **13** (0.452 g) as a yellow oil in 89% yield. Spectral analysis matched with the data reported in [69].

#### 4.3.6. *N*,*N*′-((Diselanediylbis(2,1-phenylene))bis(methylene)) bis (*N*-isopropylpropan-2-amine) (**6**)

To a solution of **13** (1.18 g, 4.37 mmol) in hexane (10 mL) cooled to −78 °C, *t*-BuLi (1.7 M in pentane, 5.1 mL) was added dropwise under argon atmosphere. The reaction mixture was stirred for 1 h and then was allowed to reach room temperature during the next 1 h. Dry THF (10 mL) was added to the reaction mixture, and the diluted solution was cooled to −78 °C when grey selenium powder was added. The mixture was allowed to warm to room temperature, quenched with 1 M HCl solution (20 mL) and extracted with diethyl ether. Ethereal layer was washed with additional 10 mL of 1 M hydrochloric acid. The combined acidic aqueous layers were alkalized by slow addition of potassium carbonate and the compound was extracted with ethyl acetate (5 × 20 mL). The resulting organic extracts were dried over magnesium sulfate, filtered and concentrated in vacuo. The crude product was purified by column chromatography (silica gel, petroleum ether-ethyl acetate 10:1) and then recrystallized from ethyl acetate/hexane to afford product (**6**) (0.754 g) as yellow crystals in 64% yield, mp = 117–118 °C [70]. ^1^H-NMR (CDCl_3_) δ = 1.09 (d, *J =* 6.6 Hz, 12H, C*H_3_*), 3.10 (sept, *J =* 6.6 Hz, 2H, C*H*), 3.86 (s, 2H, CH_2_), 7.04–7.17 (m, 2H, Ar*H*), 7.26–7.31 (m, 1H, Ar*H*) 7.70–7.74 ppm (m, 1H, Ar*H*). ^13^C-NMR (CDCl_3_) δ = 20.71, 48.33, 51.46, 126.00, 127.61, 128.59, 130.37, 132.63, 141.22 ppm; ^77^Se NMR (CDCl_3_) δ = 408.53 ppm; HRMS calculated for [C_26_H_41_N_2_Se_2_^+^] = 541.1600, found = 541.1605.

#### 4.3.7. 1-Bromo-2-(methoxymethyl)benzene (**14**)

To a solution of 2-bromobenzyl alcohol (1.97 g, 10.53 mmol) in 10 mL of THF cooled to 0 °C, sodium hydride (60% suspension in paraffin oil) (0.46 g, 11.58 mmol) was added under argon atmosphere. The mixture was stirred for 0.5 h at this temperature and 0.5 h at room temperature additionally. Methyl iodide (1.65 g, 11.58 mmol) was diluted with THF (4 mL) and added dropwise. The reaction mixture was refluxed for 4 h, then it was allowed to cool to room temperature. Saturated aqueous solution of ammonium chloride was poured into the reaction mixture, and the product was extracted with Et_2_O (3 × 30 mL). The combined organic extracts were dried over magnesium sulfate, filtrated and concentrated in vacuo. The product purified by distillation (106–108 °C/16 mmHg) in 82% yield as a colorless oil (1.74 g). ^1^H-NMR (CDCl_3_) δ = 3.47 (s, 3H, C*H_3_*), 4.53 (s, 2H, C*H_2_*), 7.15 (dt, *J =* 1.7 and 7.6 Hz, 1H, Ar*H*), 7.32 (dt, *J =* 7.6 and 1 Hz, 1H, Ar*H*), 7.45–7.57 ppm (m, 2H, ArH). Spectral analysis matched with the literature data reported in [71].

#### 4.3.8. 1-Bromo-2-(ethoxymethyl)benzene (**15**)

Sodium hydride (60% in paraffine oil, 0.538 g, 13.5 mmol) was suspended in 4 mL of THF and the suspension was cooled to 0 °C. At this temperature, a solution of 2-bromobenzyl alcohol (2.287 g, 12.2 mmol) in THF was added dropwise under argon. The reaction mixture was stirred at 0 °C for 0.5 h, then warmed to room temperature and stirred for 0.5 h. Ethyl iodide (1.46 mL, 18.3 mmol) was added dropwise and the mixture was heated under reflux for 6 h. An aqueous solution of ammonium chloride (20 mL) was poured into the mixture, and the product was extracted with diethyl ether (3 × 20 mL). The combined organic phases were washed with brine, dried over magnesium sulfate and filtered. The evaporation of the solvent followed by the distillation (120 °C/15 mmHg) afforded the product (**15**) as colorless liquid in 88% (2.32 g). ^1^H-NMR (CDCl_3_) δ =1.29 (t, *J =* 7.0 Hz, 3H, C*H_3_*), 3.63 (q, *J =* 7.0 Hz, 2H, C*H_2_C*H_3_), 4.57 (s, 2H, C*H_2_*), 7.14 (dt, *J =* 7.6 Hz, *J =* 1.6 Hz, 1H, Ar*H*), 7.32 (dt, *J =* 7.5 Hz, *J =* 1 Hz, 1H, Ar*H*), 7.51 ppm (dt, *J =*8.4 Hz, *J =* 1 Hz, 2H, Ar*H*).

#### 4.3.9. 1,2-Bis(2-(methoxymethyl)phenyl)diselane (**2**)

Compound **2** was prepared by following the procedure reported in [72] with few modifications. To a solution of 2-bromobenzyl methyl ether **14** (0.57 g, 2.83 mmol) in hexane (10 mL) stirred at −78 °C under argon atmosphere, *t*-BuLi (1.8 mL, 3.11 mmol) was added dropwise. The mixture was stirred at this temperature for 0.5 h and allowed to warm to room temperature. After the addition of THF (10 mL) and subsequent cooling to −78 °C, powder selenium (0.224 g, 2.83 mmol) was added. The reaction mixture was stirred with warming to room temperature. An aqueous solution of ammonium chloride (20 mL) was poured into the reaction mixture which was allowed to have contact with atmospheric oxygen. The product was then extracted with ethyl acetate (3 × 20 mL), the combined organic layers were washed once with brine and dried over magnesium sulfate. The drying agent was filtered off and the solvent evaporated in vacuo to give the crude product. Its purification by silica gel chromatography with eluent ether-petroleum ether 1:10 afforded 0.267 g of the compound (**2**) (51% yield) as dark yellow oil. ^1^H-NMR (CDCl_3_) δ = 3.39 (s, 3H, C*H_3_*), 4.54 (s, 2H, C*H_2_*), 7.19–7.24 (m, 2H, Ar*H*), 7.3–7.31 (m, 2H, Ar*H*), 7.75–7.76 ppm (m, 2H, Ar*H*). ^13^C-NMR (CDCl_3_) δ = 58.05, 74.77, 127.48, 128.43, 128.91, 131.81, 132.79, 138.26 ppm. ^77^Se NMR (CDCl_3_) δ = 414.63 ppm. HRMS calculated for [C_16_H_19_O_2_Se_2_^+^] = 402.9715, found = 402.9721.

#### 4.3.10. 1,2-Bis(2-(ethoxymethyl)phenyl)diselane (**3**)

To a solution of 2-bromobenzyl ethyl ether **15** (1.068 g, 4.97 mmol) in hexane (25 mL) stirred at −78 °C under argon atmosphere, 1.7 M pentane solution of *t*-BuLi (5.8 mL, 9.93 mmol) was added dropwise. The mixture was stirred at this temperature for 1 h and allowed to warm to room temperature. After the addition of THF (30 mL) and subsequent cooling to −78 °C, powder selenium (0.392 g, 4.97 mmol) was added. The reaction mixture was allowed to reach room temperature and then stirred for 18 h. An aqueous solution of ammonium chloride (50 mL) was poured into the reaction mixture which was allowed to have contact with atmospheric oxygen. The product was extracted with ethyl acetate (3 × 30 mL), the combined organic layers were washed once with brine and dried over magnesium sulfate. The drying agent was filtered off and the solvent evaporated in vacuo to give the crude product. Its purification by silica gel chromatography with eluent ether-petroleum ether 0.5:9.5 afforded 0.725 g of the compound (**3**) (68% yield) as an orange oil. ^1^H-NMR (CDCl_3_) δ = 1.26 (t, *J =*7.0 Hz, 3H, C*H_3_*), 3.56 (q, *J =* 7.0 Hz, 2H, C*H_2_*CH_3_), 4.59 (s, 2H, C*H_2_*), 7.18–7.23 (m, 2H, Ar*H*), 7.29–7.30 (m, 2H, Ar*H*), 7.75–7.77 ppm (m, 2H, Ar*H*); ^13^C-NMR (CDCl_3_) δ = 15.28, 65.82, 72.85, 127.26, 128.31, 128.79, 131.83, 132.50, 138.41 ppm; ^77^Se (CDCl3) = 411.53 ppm. HRMS calculated for [C_18_H_23_O_2_Se_2_^+^] = 431.0028, found = 431.0028.

#### 4.3.11. 2,2′-Diselanediylbis(*N*-methylbenzamide) (**8**)

To a suspension of **7 [52]** (510 mg, 1.27 mmol) in dry toluene (5 mL), thionyl chloride (0.2 mL, 2.82 mmol) was added. The resulting mixture was refluxed until no gas evolution was observable and a clear solution was formed. The unreacted thionyl chloride was evaporated in vacuo and the residue was washed with dry toluene (three times, 3 mL each). The brown oil was solubilized in dry THF (8 mL) and poured into a 3 neck round bottom flask then pure methylamine was added until a precipitate was formed. The suspension was allowed to react at room temperature for 12 h. The solid was filtrated and the filtrate was evaporated in vacuo, giving a yellow solid which was purified by column chromatography eluting with dichloromethane: methanol 99:1. A white solid was obtained and then recrystallized with EtOH (365 mg, 67%). Mp > 250 °C; ^1^H-NMR (DMSO-*d*_6_) δ: 2.78 (d, *J* = 4.37 Hz, 3H, NHC*H_3_*); 7.28 (t, *J* = 7.37 Hz, 1H, Ar*H*); 7.34 (t, *J* = 7.65 Hz, 1H, Ar*H*); 7.63 (d, *J* = 7.65, 1H, Ar*H*); 7.73 (d, *J* = 7.65, 1H, Ar*H*); 8.68 (bq, *J* = 4.37 Hz, 1H, N*H*CH_3_) ppm. ^13^C-NMR (DMSO-*d*_6_) δ: 26.89; 126.68; 128.26; 130.29; 132.28; 132.42; 133.29; 168.24 ppm. ^77^Se NMR (DMSO-*d*_6_) δ: 441.84 ppm. HRMS calculated for [C_16_H_17_N_2_O_2_Se_2_^+^] = 428.9620 found = 428.9611.

#### 4.3.12. Benzo[*d*][1,2]selenazol-3(2*H*)-one (**10**)

To a suspension of 7 [52] (175 mg, 0.44 mmol) in dry toluene (3 mL), thionyl chloride (0.2 mL, 2.82 mmol) was added. The resulting mixture was refluxed until no gas evolution was observable and a clear solution was formed. The unreacted thionyl chloride was evaporated in vacuo and the residue was washed with dry toluene (three times, 2 mL each). The brown oil was solubilized in dry THF (8 mL) and poured into a 3 neck round bottom flask then pure ammonia was added until a precipitate was formed. The suspension was allowed to react at room temperature for 12 h. The solid was filtrated and the filtrate was evaporated in vacuo giving a yellow solid which was treated with Et_2_O then filtered and recrystallized from EtOH giving the target compound as a white solid (120 mg; 71% yield). Mp = 221–223 °C; ^1^H-NMR (DMSO-*d*_6_) δ: 7.40 (t, *J* = 7.79 Hz, 1H, Ar*H*); 7.60 (t, *J* = 8.29 Hz, 1H, Ar*H*); 7.80 (dd, *J* = 0.72 and 7.77 Hz, 1H, Ar*H*); 8.03 (d, *J* = 8.29 Hz, 1H, Ar*H*); 9.18 (bs, 1H, N*H*) ppm; ^13^C-NMR (DMSO-*d*_6_) δ: 125.77; 126.39; 127.40; 127.68; 131.70; 141.56; 168.89 ppm. ^77^Se NMR (DMSO-*d*_6_) δ: 796.71 ppm. HRMS calculated for [C_7_H_6_NOSe^+^] = 199.9615 found = 199.9621

#### 4.3.13. Ethyl 2-(3-oxobenzo[*d*][1,2]selenazol-2(3*H*)-yl)acetate (**11**)

To a suspension of 7 [52] (400 mg, 1 mmol) in dry toluene (6 mL), thionyl chloride (0.47 mL, 6.5 mmol) was added. The resulting mixture was refluxed until no gas evolution was observable and a clear solution was formed. The unreacted thionyl chloride was evaporated in vacuo and the residue was washed with dry toluene (three times, 2 mL each). The brown oil was solubilized in dry THF (8 mL) and poured into a 3 neck round bottom flask then a mixture of glycine ethyl ester hydrochloride (360 mg, 2.5 mmol) and Et_3_N (1.4 mL, 10.3 mmol) was added by dropping at 0 °C. A suspension rapidly formed and was allowed to react at room temperature for 12 h. The suspension was filtered and the filtrate was evaporated in vacuo giving a yellow oil purified by column chromatography eluting with dichloromethane:methanol 99:1. A white solid was obtained and recrystallized by EtOH, giving 330 mg of target compound (66% yield). Mp = 118–120 °C. ^1^H-NMR (CDCl_3_) δ: 1.29 (t, *J* = 7.00 Hz, 3H, OCH_2_C*H_3_*); 4.24 (q, *J* = 7.00 Hz, 2H, OC*H_2_*CH_3_); 4.59 (s, 2H, NC*H2*); 7.41 (t, H = 7.37 Hz, 1H, Ar*H*); 7.60 (t, H = 7.30 Hz, 1H, Ar*H*); 7.64 (d, *J* = 8.02 Hz, 1H, Ar*H*); 8.05 (d, *J* = 7.80 Hz, 1H, Ar*H*) ppm. ^13^C-NMR (CDCl_3_) δ: 14.17; 45.62; 61.89; 123.97; 126.06; 126.27; 128.98; 132.42; 139.01; 167.84; 168.70 ppm. ^77^Se NMR (CDCl_3_) δ: 935.43 ppm. HRMS = calculated for [C_11_H_12_NO_3_Se^+^] = 285.9982, found = 285.9993.

### 4.4. Molecular Modeling

Compound **5** was sketched using the Maestro GUI (Schrödinger Release 2018-4: Maestro, Schrödinger, LLC, New York, NY, USA, 2018) and its ionization states were predicted using Epik [73] at a pH range of 7 ± 1; the state with the lowest ionization penalty was chosen for the following docking studies. The docking target structure 5DCG was downloaded from the Protein Data Bank and prepared, analogously to previously reported studies [74,75], using the Protein Preparation Wizard [76]. AutoDockTools v1.5.6 [77] was used to prepare ligand and protein input files for the docking simulations. Molecular docking simulations were performed using AutoDock Vina [54]. The search space was set as a cube (62.5 Å side) centered on the protein and including both chain A and B. Considering the pretty big search space, exhaustiveness was set to 1000. The best scoring pose (−7.7 kcal/mol) was considered as the predicted bound conformation. Molecular modeling pictures were generated using open source PyMol (The PyMOL Molecular Graphics System, Version 1.7.0.0-1, Schrödinger, LLC., New York city, NY, USA)

## 5. Conclusions

In summary, a series of selenium-containing compounds were purposely synthesized and stepwise tested for their cytotoxic properties; three out of eleven were eventually selected for an in depth biological screening meant to determine their anti-GST properties and synergism with marketed antineoplastic drugs. The enzymatic results were then investigated by computational methodologies that suggested a possible binding mode of the most interesting compound. The results presented in this manuscript may enlarge the SAR information of the diselenide and benzisoselenazolone class of compounds reported in literature. As a result of this study, we have identified compound **5** as a new lead compound endowed with a fairly potent antiproliferative activity in vitro toward MCF7 cancer cells. It was also able to potentiate the antiproliferative activity of the marketed drug cis-Pt in a dose dependent fashion. Compound **5** surely emerges as a suitable starting point for a future drug discovery campaign. In particular, given the availability of the experimental structural information, a structure-based optimization, meant to fine-tune the compound activity, will be the next step of our drug discovery program.

## Figures and Tables

**Table 1:** The IC_50_ values calculated from the dose-response curves. Cells were incubated with compounds for 48 h. All the values are given in µM.

Compound	HeLa	K562	MCF7	HUVEC
**1**	11.0 ± 6.4	4.2 ± 1.3	3 ± 1.4	3 ± 2.9
**2**	>100	62 ± 6.9	18 ± 1.4	30 ± 4.0
**3**	>100	88 ± 9.2	28 ± 1.3	160 ± 6.9
**4**	55 ± 5.8 ^1^	20 ± 1.2 ^2^	18 ± 2.1 ^1^	9 ± 1.0
**5**	20 ± 1.9 ^1^	30 ± 1.5 ^2^	15 ± 4.9 ^1^	30 ± 2.8
**8**	>100	18 ± 3.6	20 ± 1.0	21 ± 3.1
**9**	>100	85 ± 8.4	20 ± 1.8	31 ± 6.0
**10**	21 ± 5.6	22 ± 7.8	18 ± 0.9	20 ± 2.8
**11**	20 ± 4.0	32 ± 5.7	10 ± 0.9	20 ± 2.7
**Cis-Pt**	Nd ^3^	>50	Nd ^3^	15 ± 1.54
**Doxorubicin**	0.5 ± 0.01	1.1 ± 0.06	Nd ^3^	0.4 ± 0.14

^1^ During MTT assay, medium was replaced before addition of tetrazolium salt; ^2^ Determined by ATP measurement using CellTiter-Glo luminescence cell viability assay; ^3^ Not determined.
